# A Case of Transfusion

**Published:** 1879-09

**Authors:** F. W. Epley

**Affiliations:** New Richmond, Wis.


### ARTICLE II.

A CASE OF TRANSFUSION. By F. W. EPLEY, M.D., New Richmond, Wis.

Mrs. B., married, aged 37, (?) was the mother of four children, two of whom are now living, the other two having died in infancy, of a wasting disease; "they simply pined away and died without a symptom of pain or local trouble;" at least it was so stated. Mrs. B. usually enjoyed good health until her present sickness, with the exception of a "spell of ill health" when about 19 years of age. Her present sickness began about the middle of December, 1877, with an attack of what was then thought to be biliousness, from which she partially recovered, so as to be able to walk about some; soon, however she began to decline, growing anæmic, weak and languid; she seemed to be wasting away exactly as her children had done. She was treated by her home physician without effect; then went into the hands of a homœopathic physician, in whose care she remained until April 18, 1878, when I saw her for the first time, and was struck by the peculiar, almost post mortem appearance of the patient. The expression of her face was one not easily forgotten. Her eyes were glassy, sclerotic, very white; lips, gums, tongue and conjunctival membrane white and dry; lips and tongue peeling; cheeks flabby; skin dry and scaly. Has no appetite; eats a little potato and sucks the juice from a little steak; and even the small amount of food taken passes right through the canal in almost exactly the same condition in which it was taken; in fact she digests nothing—has six or seven loose discharges from the bowels daily. Complains of no pain or soreness anywhere; says there is none. Converses very plainly but slowly; sentences disconnected. Upon examination, as I placed my open hand over the epigastrium, making gentle but firm pressure, she exclaimed, "Oh, he's found it." There was no soreness to be found anywhere else, but she could bear but the slightest pressure at this point without producing a most unbearable, sickening pain. I immediately put her upon lactopeptine and bismuth, with tonics, dialyzed iron, etc., with the effect of arresting in a measure the diarrhœa, but otherwise her condition remained about the same, except that she grew daily weaker, so that, by the 27th of April, she could hardly move her hand or head. Medicine had little, if any, effect upon her. The indications plainly were to get her to make blood. This I could not do, so I decided to introduce by artificial means the much needed element. Accordingly (having read in the January number of the *Monthly Abstract of the Medical Sciences* for 1877 an article in which the hypodermic injection of blood was followed by good results) on the 27th of April I proceeded to introduce about 8 C. C. of defibrinated blood under the skin of my patient's arms, making four punctures, the last of which I tried to introduce directly into a vein, and happened to succeed — not a very easy thing to do, I have since discovered, even when the veins were well distended. She hardly felt the several punctures. An ecchymosed spot was produced at the point of each puncture, except the one which entered the vein. The following day I thought I could detect an increase in pulse rate and volume. I find recorded in my diary for that date: "Pulse much stronger, slower and fuller to-day, but she has failed otherwise. Passes urine and fæces involuntarily." Ordered vitalized phosphates added to her list of remedies, which now amounted to lactopeptine and dialyzed iron. On the 29th Dr. Otis Hoyt saw the patient with me, said he could suggest nothing except another operation soon.

May 1st. — Is delirious; takes nourishment very badly; does not arouse readily.

I would here express my regrets that my notes on the case are so brief; I had so little hope of saving or helping my patient, that I had little care about making notes. After the introduction of the first blood on the 27th, the skin seemed gradually to become more sensitive, and when, on the 5th of May, I repeated the operation, she felt the pain very acutely. The blood was taken from the arm of my student and assistant, Mr. Jackson (as, indeed, was the first), defibrinated and kept, by means of a water bath, at a temperature not less than 33° C., and was introduced by six punctures directly, or as nearly so as possible, into the vein, about 2 C. C. at each puncture. The next day there was a very noticeable increase in volume and strength in the pulse; also a slowing. From this time forward she steadily improved; the effect being rather a decided and lasting tonic or stimulant to the whole organism, than otherwise.

On the night of May 9, I was summoned in great haste, and found her suffering very acutely from phlebitis in the left lower extremity (temperature 103° F.— 39 3-5 C.—pulse 120), which gradually subsided under appropriate treatment. Afterward, during her convalescence, she had repeated attacks of local inflammations, such as pleuritis, pneumonitis, etc.; not very severe in character, but causing considerable pain at the time. Notwithstanding all this she steadily and daily improved, until, on the fifth day of the July following I took her out to ride. Has had almost constant wandering neuralgic pains the past two months, but these are subsiding now. After her first ride, she rode a little every day, and on the 14th of July she rode over to my office, a distance of about six miles.

She continued to improve until she was better, and weighed more than she had for several years. Her cheeks were rosy, lips red, eyes bright and expressive. She enjoyed good health after this until about the middle of the following December, at times her servant girl leaving her to do all the work for five or six in the family, for six weeks at a time. She was a woman of extraordinary ambition, not only in home, but in public affairs; being even during her illness the instigator of and the leading spirit in a movement by which a very neat church was built. About the middle of December, after having overworked herself on a piece of heavy sewing, she became nauseated, soon vomited quantities of biliary matter, mucus, etc.; skin became icteric, and she began to decline. She visited me at my office several times, and I prescribed various remedies, but all to no purpose; she gradually grew worse, and on the 30th of December I saw her at her residence, flat on her back, vomiting large quantities of biliary matter, mucus, etc. Had no appetite, sleep very much disturbed, and was unable to raise her head without nearly fainting. Prescribed strychnia, Gm. .0006, 3 times a day.

Jan. 1. Seems better in every respect, except color fading from mucous membranes. Vomits nothing, and is hungry. Sordes gathering on teeth and gums.

Jan. 3. Has vomited a large amount of ropy mucus and undigested food during the last twenty-four hours. Has no pain anywhere, but cannot have her head raised on account of vertigo. Prescribed pancreatine, and nourishing aliment, etc., continued.

Jan. 5th.—Pulse 114, temperature 38 8-10; sleeps badly and is nauseated. Raises no mucus nor bile. Movements from bowels pasty but regular. Complains of very bitter taste in mouth, especially after taking milk. Began menstruating; discharge scanty and light colored. Has no appetite; takes from one to three raw eggs a day; does not like to take milk on account of the very bitter taste it produces; constant, distressing nausea, controlled by small doses of bism. subnit., and morphia when required. Eye bright, sclerotic, snowy white, like the "celestial eye," described by good old Professor Freer.

Jan. 8th.—Great tenderness on hepatic region and right mesenteric glands; almost faints at a touch in that locality. Is vomiting again large quantities of yellow looking matter, which, however, do not respond to the tests for bile. Bowels constipated; ordered enema, to be followed shortly by beef tea per rectum, with counter irritants to epigastrium, and acid nitro-muriatic .12 C. C. every third hour; and stopped pancreatine and strychnine.

Jan. 9th.—Pulse 108, temperature 38 3-10° C., tongue pale, skin on lips dry and peeling; is drowsy all the time. Has beef tea regularly per rectum, and fluid nourishment per stomach, all she can dispose of.

Jan. 11th.—Has failed rapidly since I last saw her; seems stupid; sensibilities benumbed; eyes dull and hair dry. Vomited, last night, a large quantity of horrible-looking stuff—masses of ropy mucus, slime and a substance resembling chopped meat (which she has not eaten in nearly a week). Pulse, 120; temperature, 38.8. She is sinking so fast I shall transfuse to-morrow.

Jan. 12th. — Transfused 10:30 a. m. Before the operation: pulse, 116, quick, weak and thready; temperature, 39.2° C.; respiration labored and sighing; sentences broken; lips, tongue and gums white, no signs of blood in them; bowels constipated; has no appetite. Passed a pretty good night, but when awakened, complained of an indescribable feeling of oppression, exclaiming in broken accents, "Oh dear! oh dear! what shall I do." Nausea constant and distressing. No salivary secretion to moisten food with, so she rolls it out of her mouth, after sucking the moisture from it. Lips covered by thick, dead skin, which peels off, leaving a pale, shriveled fold, with only the faintest tinge of a dirty pink color.

*Operation.* — 150 C. C. of blood was taken from the radial vein of Dr. C. F. King, who assisted me in the operation, defibrinated and kept, by means of a water bath, at a temperature not exceeding 36° C. The radial veins of each arm of the patient, respectively, having been previously partially anæsthetized locally by means of the ether spray, were opened, the canula of an instrument made by Sharp & Smith, of Chicago, expressly for this purpose, and in the form of a very large hypodermic syringe with blunt points, was inserted, and 4 C. C. injected into each vein, only 8 C. C. in all being used. This would seem a very small amount, but I am convinced that it was all the patient could stand at once, or at least, that more would have been worse than useless, for the result left nothing to be desired, and was truly marvelous. Thirty minutes after the operation the pulse fell to 98 — full, soft and regular. A sensation of warmth and tingling was felt extending to the ends of the fingers and toes. We left her one hour after the operation, calling for something to eat.

Jan. 13th. — Passed a first-rate night; slept well; had none of the feelings of oppression on waking experienced just before the operation. Felt strange sensations of prickling in arms, legs and fingers. Strange phenomena of vision occurred during the afternoon and night after the operation — "knitting needles in the air, spools of thread rolling around the room, etc." While making my visit to-day, being momentarily out of the room, "a perfectly transparent straw" made its appearance before her, whose ends she could point out, and could bring her finger down upon it from above; said she "toyed with it for some little time; knew it was not a straw or anything else tangible, but regarded it as very singular." Pulse 98, soft and full; temperature, 38.8 C. Lips cleaned off, and show considerable color. Respiration easy. Talks with some vigor.

Jan. 14th. — Sat up over an hour to-day, with no disagreeable symptoms whatever. Appetite good. Relishes food well. Wandering pains appear, as after the first operation, nine months ago. Pulse 90; temperature, 38. Respiration normal. Voice strong and steadier, though a little tremulous yet. Disturbances of vision abating, but still exist.

Jan. 16th. — Some headache to-day. Pulse 90, and good; temperature, 37.3. Bowels moved by enema. Cheeks flushed; lips quite red. Sat up two hours to-day without fatigue.

Jan. 18th. — Sat up most of the day. Feels well; sleeps well; has good appetite; talks well, and feels strong. Pulse 90, full and strong; temperature, 37.7 C. Veins quite full and dark.

Jan. 20th. — Has sat up most of the day. Veins full; circulation splendid. Pulse, 90; temperature not taken. Eats and sleeps well. Nothing abnormal, except slight stiffness of joints.

Specimens of blood were taken at intervals, and sent to Prof. I. N. Danforth for examination; one just before operation and one 24 hours after.

From this date — Jan. 18th — she went on making a much more rapid recovery than before. But, unfortunately, her ambition domineered over her judgment. Having been twice so near the grave and recovered, she began to think she was not born to die easily, and after three months more of health, she exposed herself to the cold and fatigue, drove a vicious, awkward horse, which became frightened and backed her down an embankment into a ditch — frightening her badly, and causing severe nervous shock, sending her into another decline, into the hands of homœopathy, and the grave, on the 16th of April, 1879.

The autopsy was made 20 hours after death, and specimens of the result forwarded to Prof. Danforth on the following morning.

NEW RICHMOND, WIS., May 30, 1879.

---

The morbid specimens sent by Dr. Epley presented no marked lesions. The surface of the gastric mucous membrane was here and there eroded, and was everywhere extremely pale and bloodless; the same is true of the section of small intestine. The right kidney was pale and slightly granular. These several appearances were probably due to absence of assimilative power or starvation, rather than to the ravages of any active pathological process. There is a marked contrast between the microscopic appearance of the red corpuscles before and after the last transfusion. Before the transfusion they appear shriveled, pinched and irregular at their edges; a few days after the transfusion they are comparatively plump and full. There is also a slight difference in the relative number of the white corpuscles in the two specimens, the last one presenting a notably less number than the one drawn previous to transfusion. The case seems to present a strong argument in favor of transfusion in instances of extreme anæmia coupled with mal-assimilation.

